# Editorial: DADA2 and other monogenic vasculitides

**DOI:** 10.3389/fimmu.2022.1108853

**Published:** 2022-12-08

**Authors:** Pui Y. Lee, Ezgi D. Batu, Seza Ozen

**Affiliations:** ^1^ Division of Immunology, Boston Children’s Hospital, Harvard Medical School, Boston, MA, United States; ^2^ Department of Pediatric Rheumatology, Hacettepe University, Ankara, Türkiye

**Keywords:** deficiency of adenosine deaminase 2 (DADA2), STING-associated vasculopathy of infancy (SAVI), monogenic vasculitis, autoinflammatory diseases, ADA2

Systemic vasculitis broadly defines a heterogenous group of inflammatory diseases that primarily affect blood vessels (1). The etiology of most systemic vasculitides remains unknown and the forms of vasculitis in children and adults are not the same. Among primary vasculitides, IgA vasculitis and Kawasaki disease are common in young children, whereas some such as Takayasu arteritis and anti-neutrophil cytoplasmic antibodies (ANCA)-associated vasculitis are more common in adults. The landmark discoveries of deficiency of adenosine deaminase 2 (DADA2) and STING-associated vasculopathy of infancy (SAVI) redefined the paradigm of systemic vasculitis (2–4). These monogenic forms of secondary vasculitis often manifest early in life and inflict devastating consequences in children and adults (5). While the causal genes for DADA2 and SAVI are defined, we have much more to learn about the complex pathophysiology of these conditions. In the Research Topic *“DADA2 and other Monogenic Vasculitides*,” we aim to advance the pathophysiology, diagnostic evaluation, and treatment for these intriguing yet potentially fatal diseases.

## An overview of monogenic vasculitides

Review articles by Signa et al. and Wobma et al. provide excellent summaries of DADA2 and SAVI, respectively. Although the precise mechanism linking defective ADA2 function to the plethora of manifestations in DADA2 remains unclear, Signa et al. synthesize the abundant evidence pointing to abnormalities in both innate immunity and adaptive immunity. Aberrant monocyte polarization and neutrophil extracellular traps are potential explanations for the excess tumor necrosis factor (TNF) production (4, 6). Recent studies also suggest dysregulation of type I interferons (IFN-I) in DADA2 (7–9), although the role of these antiviral cytokines awaits further clarification.

Wobma et al. review the stimulator of interferon genes (STING) axis of IFN-I production and discuss how dysregulation of this pathway leads to immune activation and progressive loss of tolerance. In addition to SAVI, the authors discuss the biology of other monogenic interferonopathies and share their insight on how investigations of these rare diseases inform our understanding of more common autoimmune diseases.

## Expanding the phenotype spectrum of DADA2

One of the most intriguing aspects of DADA2 is the broad spectrum of clinical manifestations (10, 11). Barron et al. provide a comprehensive update on 60 patients with DADA2 followed at the U.S. National Institute of Health. Adding to the notion that clinical manifestations of DADA2 can be categorized by vasculitic/inflammatory features, hematologic defects, and evidence of immunodysregulation, the authors note that most patients display significant overlaps between these phenotypic categories. The study also describes the impact of coronavirus disease 2019 (COVID-19) on patients with DADA2 as well as the safety of COVID-19 vaccines.

Secondary hemophagocytic lymphohistiocytosis (HLH), also known as macrophage activation syndrome, is rarely described in DADA2, Drago et al. describe a patient with recurrent HLH following parvovirus infection and varicella zoster virus reactivation as early features that eventually led to a diagnosis of DADA2. This report raises awareness for secondary HLH as a manifestation of DADA2 and illustrates the potential benefits of intravenous immunoglobulin therapy for this complication.

The genotype-phenotype correlation is another unique feature of DADA2: patients with vasculitis/systemic inflammation usually possess missense mutations in *ADA2* with residual protein function while patients with bone marrow failure typically have missense variants with complete elimination of protein function or predicted loss-of-function variants (i.e. insertions/deletions with frameshift or nonsense variants) (12, 13). Barzaghi et al. offer their insight through a rare opportunity to study identical twins with DADA2. Supporting the connection between genotype and disease phenotype, the twin sisters share almost identical features. The variable age of symptom onset and disease severity suggest a role of environmental factors in the pathophysiology of DADA2.

## A new method to measure ADA2

DADA2 is confirmed by the finding of biallelic pathogenic *ADA2* variants but not all variants are readily detectable by standard sequencing technology (see case report by Barzaghi et al.) (14). As a complementary approach to genetic testing, plasma ADA2 activity can also establish the diagnosis of DADA2. Traditional methods used to measure ADA2 activity include spectrophotometric assays that quantify the release of ammonia from the deaminase reaction and high-performance liquid chromatography assays that detect the products (i.e. inosine and hypoxanthine) (15–17). Because ADA1 catalyzes the same biochemical reaction, ADA1 inhibition is required to determine the specific activity of ADA2.

Luo et al. described a new method of quantifying ADA2 based on enzyme-linked immunosorbent assay. Using plate-bound polyclonal antibodies to capture ADA2, this method allows downstream quantification of ADA2 protein levels and enzymatic activity. In addition to validating this approach as a diagnostic test for DADA2, the authors explored the use of ADA2 levels as a biomarker of large granular leukocyte leukemia as well as head and neck cancer.

## Treatment options for DADA2

Tumor necrosis factor inhibitors (TNFi) are the standard of care in patients with DADA2 to prevent strokes and treat vasculitic/inflammatory manifestations (18, 19). Barron et al. note that among 24 patients with a history of one or more strokes (76 events in 3622 patient months prior to therapy), no strokes were observed in 2027 patient months after initiation of TNFi. However, TNFi are typically ineffective for the hematologic manifestations and may increase infection risk in patients with immunodeficiency (12).

Hashem, Meyts and colleagues have led the way in establishing allogeneic hematopoietic cell transplant (HCT) as a curative option for DADA2 patients with severe hematologic involvement, immunodeficiency and refractory vasculitis (20, 21). In their review article, Hashem et al. provide a comprehensive discussion of approaches to HCT in DADA2 including disease-specific considerations, barriers to successful engraftment, post-HCT complications, and clinical outcomes of transplanted patients.

Lastly, Hong et al. illustrate the potential use of lentiviral vector-mediated gene transfer to restore wildtype ADA2 expression. Through a series of ex vivo studies, the authors demonstrate successful restoration of ADA2 protein production and reversal of abnormal cellular phenotypes in ADA2-deficient cells.

## Concluding remarks

Less than a decade into the discovery of DADA2 and SAVI, we have made significant advances in understanding monogenic vasculitides. By consolidating the current knowledge and presenting new research directions, the collection of articles in this Research Topic brings us another step closer to curing these potentially fatal diseases.

## Author contributions

PL, EB and SO were editors of the Research Topic and drafted this editorial. All authors contributed to the article and approved the submitted version.
